# Vector competence of anthropophilic mosquitoes for a new mesonivirus in Senegal

**DOI:** 10.1080/22221751.2020.1730710

**Published:** 2020-02-28

**Authors:** Alioune Gaye, Moussa Moïse Diagne, El Hadji Ndiaye, Marie Henriette Dior Ndione, Martin Faye, Cheikh Talla, Gamou Fall, Yamar Ba, Diawo Diallo, Ibrahima Dia, Pascal Handschumacher, Ousmane Faye, Amadou Alpha Sall, Mawlouth Diallo

**Affiliations:** a Unité d’Entomologie Médicale, Institut Pasteur de Dakar, Dakar, Sénégal; b Pole de virologie, Unité des Arbovirus et virus de Fièvres Hémorragiques, Institut Pasteur de Dakar, Dakar, Sénégal; c Université Cheikh Anta Diop de Dakar, Dakar, Sénégal; d Aix Marseille Univ, INSERM, IRD, UMR SESSTIM, Sciences Economiques & Sociales de la Santé & Traitement de l'Information Médicale, Marseille, France; e Epidemiology of infectious diseases unit, Institut Pasteur de Dakar, Dakar, Senegal

**Keywords:** Vector competence, Dianke virus, *Culex*, *Aedes*, *Anopheles*

## Abstract

The mesoniviruses (MESOVs) belong to the newly described Mesoniviridae family (Order: Nidovirales). They have never been reported in Senegal until recently during a study in arbovirus emergence with the detection of a new species of MESOV named *Dianke virus* (DKV) from common mosquitoes from eastern Senegal. Actually, their vector competence for this newly described DKV is unknown. We, therefore, estimated the vector competence of *Culex tritaeniorhynchus*, *Culex quinquefasciatus*, *Aedes aegypti*, and *Anopheles gambiae* mosquitoes collected in Senegal for DKV using oral infection. Whole bodies, legs/wings, and saliva samples were tested for DKV by RT–PCR to estimate infection, dissemination, and transmission rates. The infectivity of virus particles in the saliva was confirmed by infecting C6/36 cells. Virus transmission rates were up to 95.45% in *Culex tritaeniorhynchus*, 28% in *Cx. quinquefasciatus* and 9.09% in *Aedes aegypti*. Viral particles in the saliva were confirmed infectious by C6/36 cell culture. *An. gambiae* was able to disseminate DKV only at 20 days post-infection. This study shows that *Culex* mosquitoes are more competent than *Ae. aegypti* for DKV, while *Anopheles gambiae* is not likely a competent vector.

## Introduction

Until 2011, the Nidovirales was known as an order of positive-sense single-stranded RNA (ssRNA+) viruses that included three families: the Arteriviridae, consisting of small-genome nidoviruses (12.7–15.7 kb), the Coronaviridae and the Roniviridae, both consisting of large-genome nidoviruses (26.3–31.7 kb). The discovery of Nam Dinh [1] and Cavally [2] viruses in Vietnam and Côte d’Ivoire, respectively, resulted in the addition of a fourth family named the Mesoniviridae, in reference to their medium-sized genomes (about 20 kb) [3]. The viral particles are enveloped, 60–80 nm diameter spheres with club-shaped surface spikes [4]. Their 20 kb genome is generally organized as ORF1a-ORF1b-ORF2a-ORF2b-ORF3a-ORF3b-ORF4 [5]. ORF1a and ORF1b are predicted to encode two polymerase polyproteins (pp), while the ORFs in the 3’-end encode structural proteins such as the spike (S) glycoprotein (ORF2a), the nucleocapsid (N) protein (ORF2b), and two proteins with membrane-spanning regions (ORF3a and -3b) [1,2].

Mesoniviruses are considered as insect-specific viruses (ISVs) as they were isolated only from mosquito pools or cell lines with no detection in vertebrates [1,2,5,6]. ISVs were also found in sandflies [7] and chironomids, indicating that they can also infect other dipterans. An increasing number of arthropod-specific viruses (ASVs) are being discovered in haematophagous arthropods over the world [8], and there is a growing interest on mesoniviruses interactions with viruses of public health concern to develop strategies that could curb arbovirus transmission through dual infection and competition, as implemented with the *Wolbachia* mediated strategy [9]. Some studies suggested for instance that ISVs negatively affect the fitness of West Nile (WNV) [10–12] and dengue (DENV) [13,14] viruses in the mosquito, while other studies demonstrated that there is no effect [15] or even an enhancing effect [11]. However, there are still few studies on mesoniviruses phenotypic behaviour in mosquito vectors.

A new mesonivirus species was recently reported in Senegal [16]. The so-called Dianke virus (DKV) exhibited a wide host range within mosquito populations. Some of them are important vectors for arboviruses or *Plasmodium* species.

The aim of this study was to characterize DKV fitness in four different anthropophilic mosquito species of public health concern. This work paves the way toward future investigations on potential interactions between DKV and other vector transmitted pathogens and brings forward the possibility of a second host implied in the DKV life cycle.

## Materials and methods

### Mosquito species

We used four mosquito species representing *Culex*, *Aedes*, and *Anopheles* genus. *Cx. quinquefasciatus* and *Cx. tritaeniorhynchus* were collected in Barkedji (15°16′50.242” N, 14°51′54.751” W). *Aedes aegypti* and *An. gambiae* were collected in Dakar (14°43'29” N, 17°28'24” W). These species were chosen because of their abundance, anthropophilic behaviour, and association with DKV and other previously described mesoniviruses in the field [2,16].

Larvae and pupae were collected from the field. Adults were reared in the laboratory at 27 ± 1°C and relative humidity of 70–75%, with a 12 h photoperiod. Females (F0) were fed several times on guinea pigs to obtain F1-generation eggs. These were hatched, and the larvae were reared under the conditions described above to obtain the F1 adults used in this study. Mosquitoes were fed on a 10% sucrose solution.

### Virus strain and preparation of the stock

The DKV isolate (Genbank accession number: MN622133) was passaged twice in C6/36 cell cultures (Table 1). An additional passage into monolayers C6/36 cells was performed as previously described [16] to obtain the viral stock used to infect mosquitoes. Briefly, Continuous cells lines, initially provided by the American Type Culture Collection (ATCC), were cultured in Leibovitz-15 (L-15) medium (GibcoBRL, Grand Island, NY, USA) supplemented with 10% foetal bovine serum (FBS) (Gibco BRL, Grand Island, NY, USA) and penicillin- streptomycin (Sigma, GmBh, Germany) and 10% of Bacto™ Tryptose Phosphate Broth (*Becton, USA*) and maintained in 25-cm^2^ tissue culture flasks. The medium was removed from the flasks before adding of 150 µl of the supernatant of the virus directly into monolayers C6/36 cells. After 1 h of incubation at room temperature, infected cells were recovered by 5 ml of L-15 medium supplemented with 5% FBS and incubated at 28°C until cytopathic effect (CPE). After harvesting, infected cells supernatants were aliquoted, frozen at −80°C, and used as viral stocks for mosquito infection. The virus stock was prepared and titrated using C6/36 cell lines. As plaque formation could not be observed on C6/36 cells, viral titres were evaluated using a pan Mesonivirus RT-qPCR assay previously used for the DKV detection in field samples [16].

**Table 1.** Virus strain used in this study.

Strain	GenBank accession number	Collection place	Original host	Passage number on cells	Collection date
ArD270551	MN622133	Barkedji (Senegal)	*Culex poicilipes* (Mosquito pool)	2	2014

After preparation of stocks on cells, viral titre of 1.6 × 10^7^ RNA copies/ml was obtained and used for mosquito infections.

### Mosquito oral infections procedure

Three- to five-day-old F1 generation female mosquitoes were placed into 0.45 L cardboard cages and starved for 48 h before being allowed to take an infectious blood meal via an artificial feeder, as previously described [17,18] using mouse skins as membranes. Mosquitoes were fed on an infectious blood meal containing 33% of washed rabbit erythrocytes and 33% of viral suspension supplemented with 33% of a mixture of FBS, adenosine triphosphate (ATP) to a final concentration of 0.005 M as a phagostimulant and a solution of 10% sucrose. Mosquito feeding was limited to 60 min. Afterwards, blood meals were stored at −80°C to later measure the number of DKV RNA copies per ml using the pan Mesonivirus RT-qPCR assay (Table 2). The mosquitoes were cold-anaesthetized to select fully engorged individuals (non-engorged females were discarded), which were transferred to cardboard containers and subsequently maintained in an incubator at 27 ± 1°C, 70–75% relative humidity, and a 12 h day/night cycle. Mosquitoes were given ad libitum access to a 10% sucrose solution.

**Table 2.** Blood meals titres at the end of each mosquito feeding.

Mosquito species	Blood meals titers (number of RNA copies/mL)
***Ae. aegypti***	6.2 × 10^4^
***An. gambiae***	1.6 × 10^5^
***Cx. tritaeniorhynchus***	1.7 × 10^5^
***Cx. quinquefasciatus***	9.8 × 10^4^

### Virus detection

At 1, 2, 3, 4, 5, 6, 7, 10, 15, and 20 days post-infection (dpi), samples of *Ae. aegypti* and *An. gambiae* were randomly collected. The low sample sizes of engorged females of *Culex* mosquitoes led us to test *Cx. tritaeniorhynchus* at 7, 10, and 15 dpi and *Cx. quinquefasciatus* only at 15 dpi. Mosquitoes were cold-anaesthetized, and their legs and wings removed and transferred individually into separate tubes. Saliva was collected by inserting the proboscis of each mosquito into a capillary tube containing 1–2 μl of FBS for 30 min. After salivation, each mosquito body (whole body except legs and wings) and saliva sample was placed in a separate tube. Detection of DKV in the mosquito body without infection of the legs, which contain haemolymph, is an indication of a non-disseminated infection limited to the midgut, whereas the presence of virus in both the mosquito body and legs indicates a disseminated infection with virus in the haemocoel. The mosquito bodies, legs/wings, and saliva were stored separately at −80°C until real-time RT–PCR could be performed. The infectiousness of positive saliva samples was tested with C6/36 cell cultures.

The samples were crushed and homogenized in 500 μl of L-15 cell culture medium (GibcoBRL, Grand Island, NY, USA) containing 20% FBS and were then centrifuged for 10 min at 7500 rpm at 4°C to separate virus supernatant and debris. For real-time PCR, 100 μl of supernatant was used for RNA extraction using the QIAamp Viral RNA Extraction Kit (QIAgen, Heiden, Germany), according to the manufacturer’s protocol. The RNA was amplified using ABI Prism 7000 SDS Real-Time apparatus (Applied BioSystems, Foster City, CA, USA) with the QuantiTect kit (QIAgen) and the pan Mesonivirus system as previously described [16]. The reaction mixture consisted of 5 μl of extracted RNA, 10 μl of buffer (2 X QuantiTect Probe), 6.8 μl of RNase free water, 1.25 μl of each primer, forward and reverse, 0.5 μl of probe, and 0.2 μl of enzymes to a total volume of 25 μl. The cycling conditions were RT step at 50.0°C for 10 min, at 95.0°C for 15 min, and 40 cycles of 15 s at 95.0°C and 1 min at 60°C.

### Infectiousness of positive saliva samples

The infectiousness of positive saliva samples after amplification on a mosquito species was tested with C6/36 cell culture in 25 cm^2^ flasks. The positive saliva samples were diluted in a 1:10 ratio in cell culture medium containing 10% FBS before filtration. Once cells reached a confluence of approximately 70%, the medium was discarded and 200 μl of diluted positive saliva samples were inoculated into monolayer C6/36 cells as described above. The flasks were gently agitated every 10 min during incubation to enhance viral dispersion. After 1 h, 5 ml of L-15 medium (5% FBS, 5% tryptose phosphate, 1% glutamine, 1% penicillin–streptomycin, and 0.05% amphotericin B) was added, and the infected cells were incubated (for 3 days) and then harvested after CPE observation. The medium was then removed and centrifuged for 5 min at 10,000 rpm and 4°C. The supernatant was collected and stored at −80°C until analyses. The detection and quantification of virus was performed using the pan Mesonivirus RT-qPCR assay. The presence of more viral particles in post-infection cultures than in saliva was taken to indicate infectiousness.

### Binary logistic regression model

The effects of mosquito species and time after exposure (dpi) on each (i) infection, (ii) dissemination and (iii) transmission phenotype have been assessed using a binary logistic regression model. Possible interactions between independent factors were tested in the model, and likelihood ratio tests comparing models with and without the interaction term were used to estimate the significance of the interaction. The time post-exposure was treated as a continuous variable and *Ae. aegypti* the main arbovirus vector has been set up as reference compared to other species. The significance of risk factors was determined by calculating Odds ratio (OR) and 95% confidence interval (CI). N represents the number of mosquitoes tested.

### Data analysis

Infection (number of positive bodies/total number of mosquitoes tested), dissemination (number of infected legs and wings/total number of infected bodies), and transmission (number of positive saliva/total number of infected legs and wings) rates were calculated for each species at each dpi. Rates were compared using Fisher’s exact test. Differences were considered statistically significant at *P* < 0.05. Statistical tests were performed using R v. 2.15.1 (R Foundation for Statistical Computing, Vienna, Austria).

## Results

**Figure 1.**
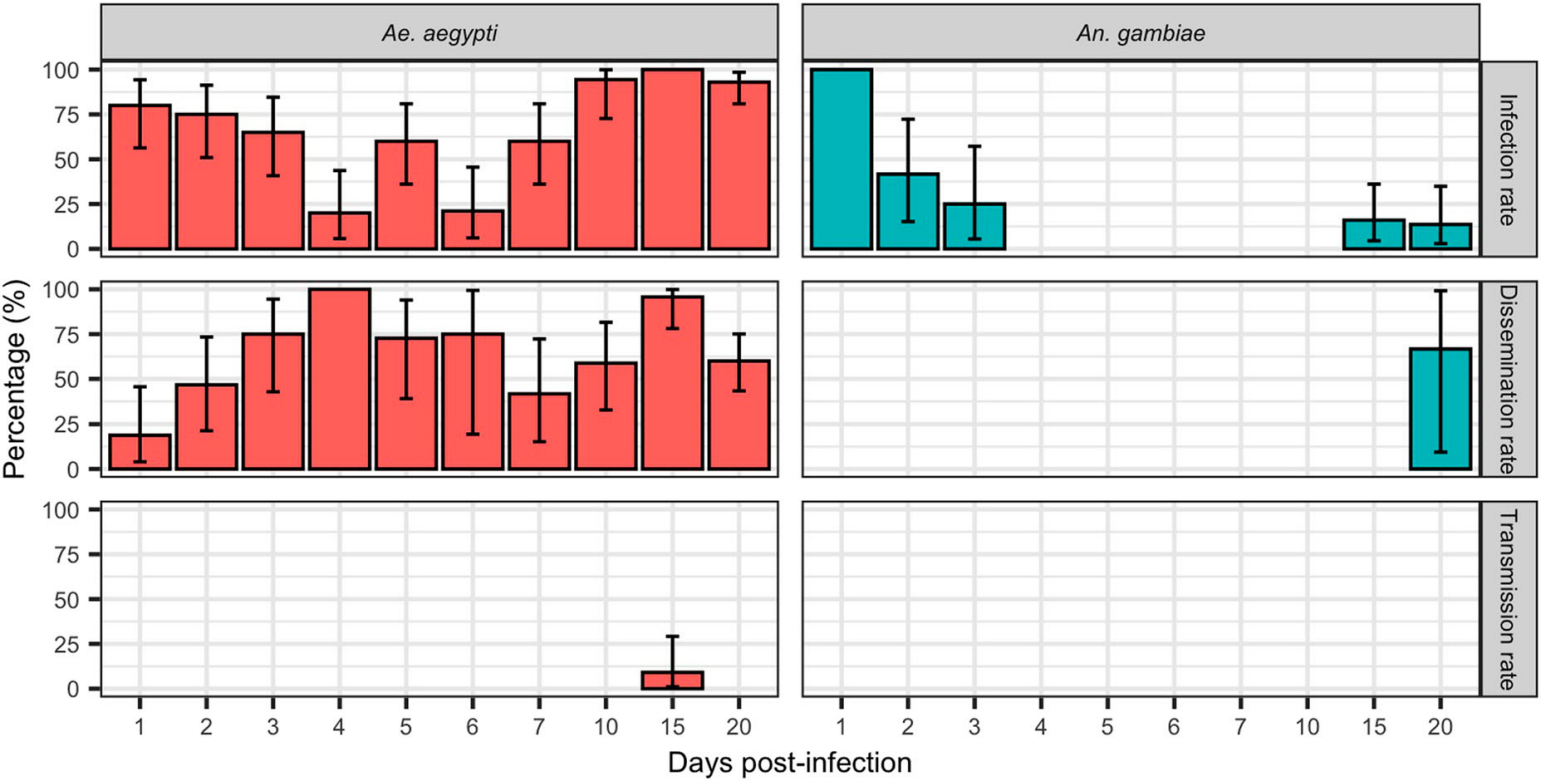
Infection, Dissemination and Transmission rates at 1, 2, 3 … 20 dpi for *Ae. aegypti* and *An. gambiae* orally exposed to DKV. Errors bar represent the upper limits of the 95% confidence intervals.

**Figure 2.**
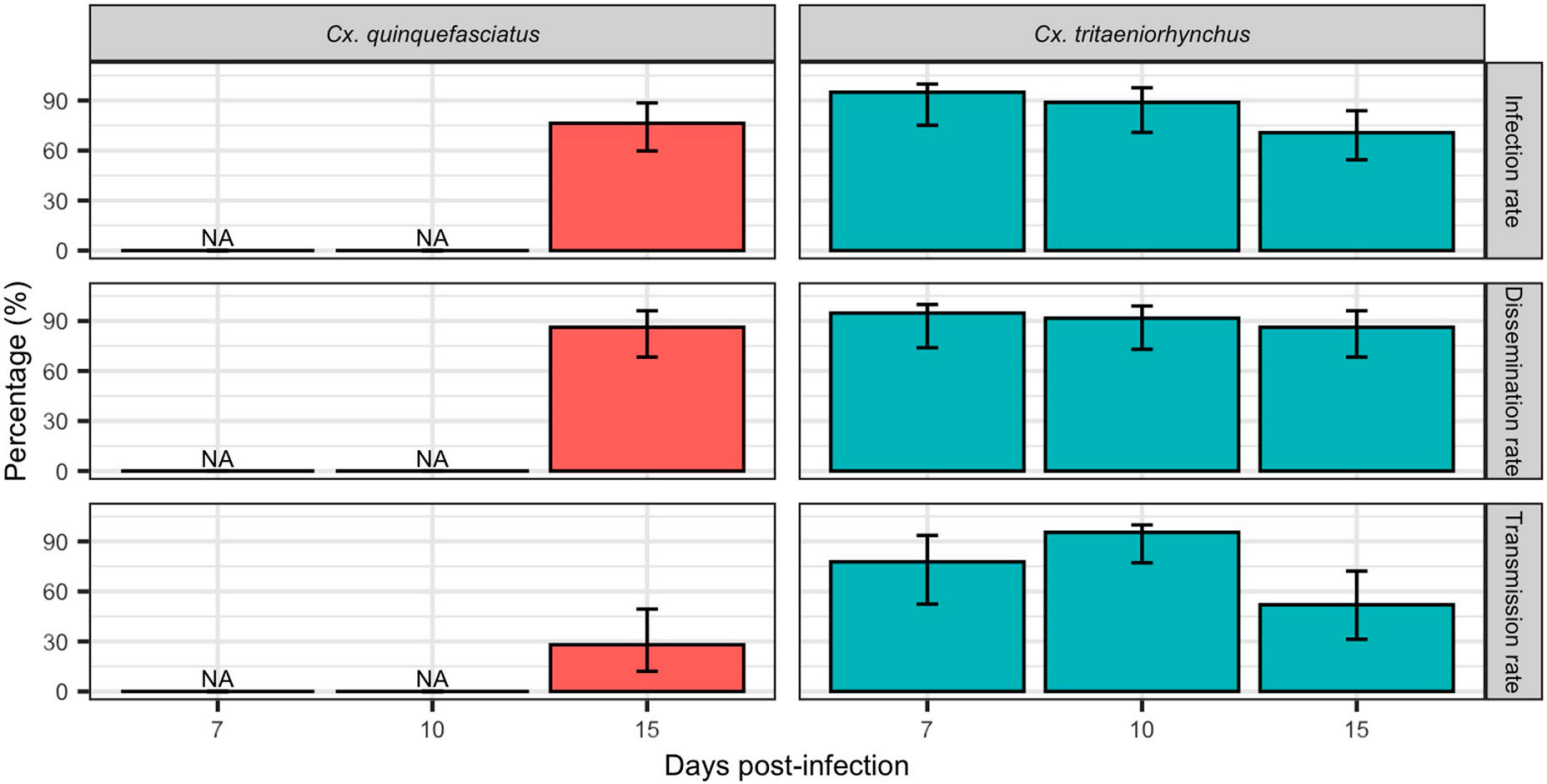
Infection, Dissemination and Transmission rates at 7, 10 and 15 dpi for *Cx. tritaeniorhynchus* and at 15 dpi for *Cx. quinquefasciatus* orally exposed to DKV. Errors bar represent the upper limits of the 95% confidence intervals.

A total of 223 *Ae. aegypti*, 131 *An. gambiae*, 88 *Cx. tritaeniorhynchus*, and 38 *Cx. quinquefasciatus* were tested. Our results (Figures 1 and 2) showed that, except for *An. gambiae*, all species were highly susceptible to infection by DKV.

Infection rates of DKV in *An. gambiae* (Figure 1) decreased after the first dpi to 0% at 4 dpi. Recrudescence was observed at 15 and 20 dpi in infected *An. gambiae* with infection rates of 16% and 13.63%, respectively. Even if *An. gambiae* developed a disseminated infection at 20 dpi, no transmission was observed.

*Ae. aegypti* was highly susceptible to infection with DKV strain. Infection rates again initially decreased after the first days, before a further increase at 6 dpi to reach 100% by 15 dpi. Dissemination was high and increased between the first to fourth dpi. *Aedes aegypti* were able to transmit DKV at 15 dpi (9.09%).

We limited our subsequent experiments to sample *Cx. tritaeniorhynchus* at 7, 10, and 15 dpi and *Cx. quinquefasciatus* at 15 dpi because of the limited number of engorged females obtained with these species not allowing testing samples at all dpi as previously.

*Culex tritaeniorhynchus* and *Cx. quinquefasciatus* were highly susceptible to infection and were able to transmit DKV. After 7 dpi, transmission rate in *Cx. tritaeniorhynchus* reached 77.77%, 95.45% at 10 dpi and 52% at 15 dpi. For *Cx. quinquefasciatus* transmission rate was 28% at 15 dpi and comparable to those for *Cx. tritaeniorhynchus* (Fisher’s exact test: *P* = 0.1).

**Figure 3.**
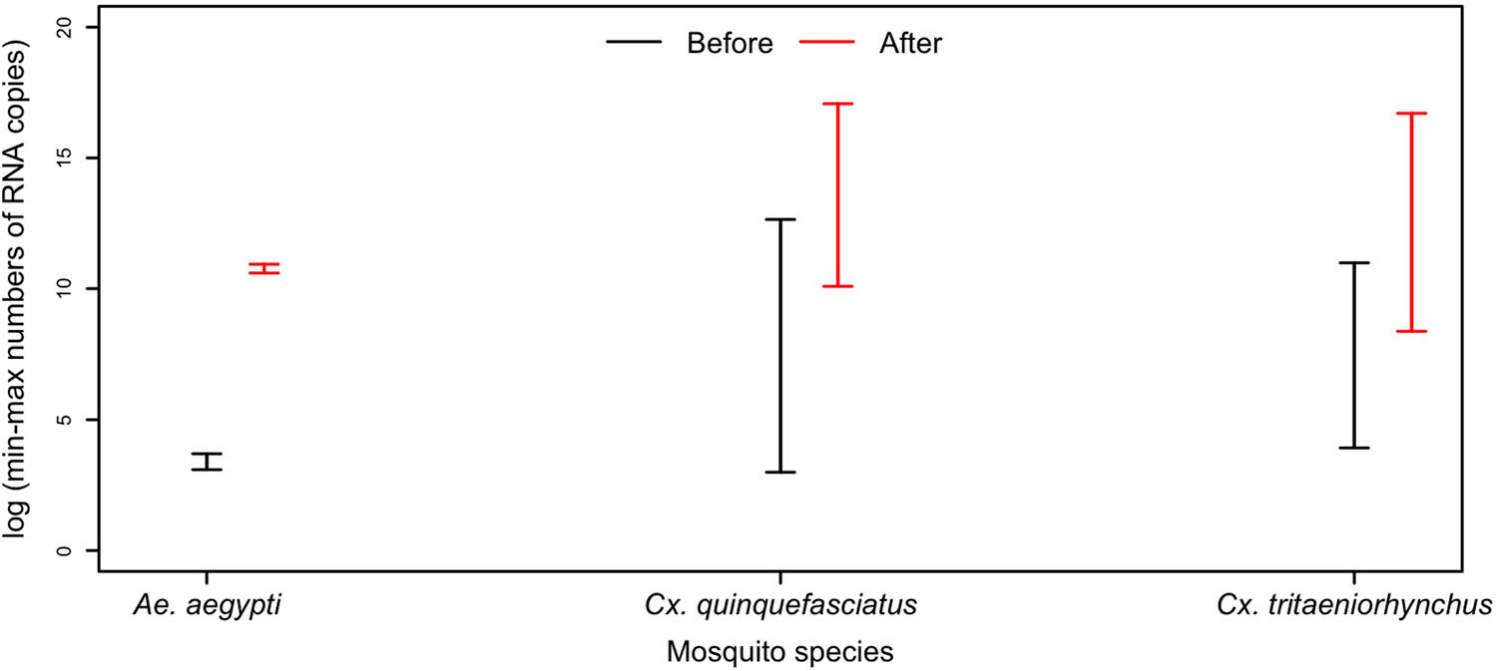
The intervals (min-max) of numbers of RNA copies of DKV in saliva (black line) and post-infection C6/36 cells cultures (red line).

All positive saliva samples induced CPE in C6/36 cells. The presence of infectious viruses in saliva was further revealed by a higher virus RNA load after amplification of C6/36 cells (Figure 3).

In binary logistic model, *Cx. quinquefasciatus* was removed from the analysis in infection and dissemination; it was only followed on single time point (at 15 dpi) which does not allow assessing day exposure effect and species-days interaction. Each additional day exposure for *Ae. Aegypti* is associated with an increased chance of getting infected (OR = 1.14, *P* < 0.001).

Concerning the effect of mosquito species *Cx. tritaeniorhynchus* is more likely to become infected than *Ae. aegypti* (*P* < 0.001).

**Figure 4.**
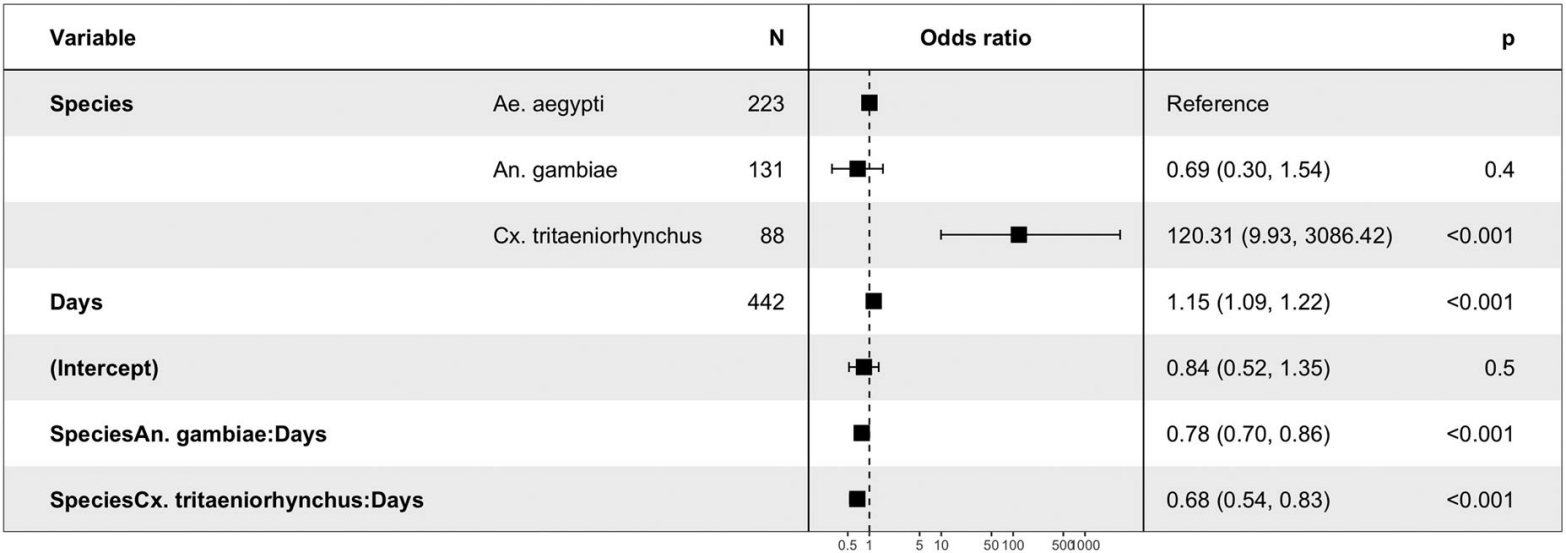
The risk factors for mosquito infection.

The exposure time (days) decreases the chance of infection for *Cx. tritaeniorhynchus* and *An. gambiae* (OR = 0.68, *P* < 0.001; OR = 0.89 (1.14*0.78), *P* < 0.001 respectively) (Figure 4).

**Figure 5.**
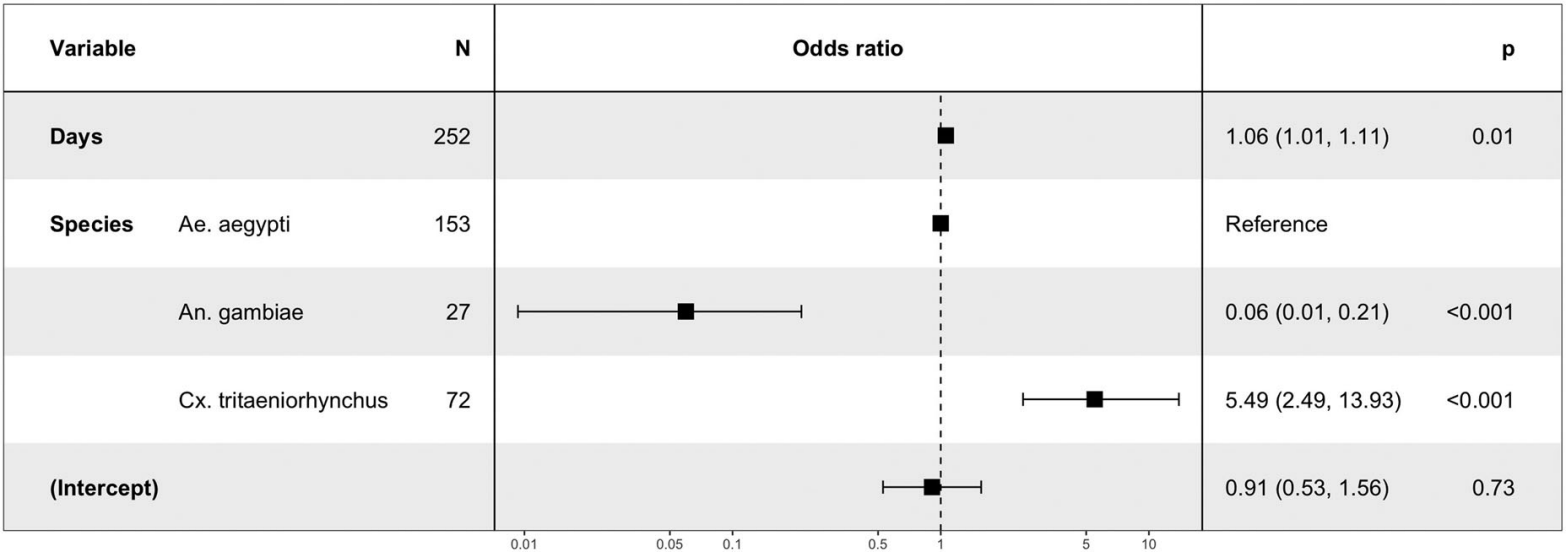
The risk factors for mosquito dissemination.

**Figure 6.**
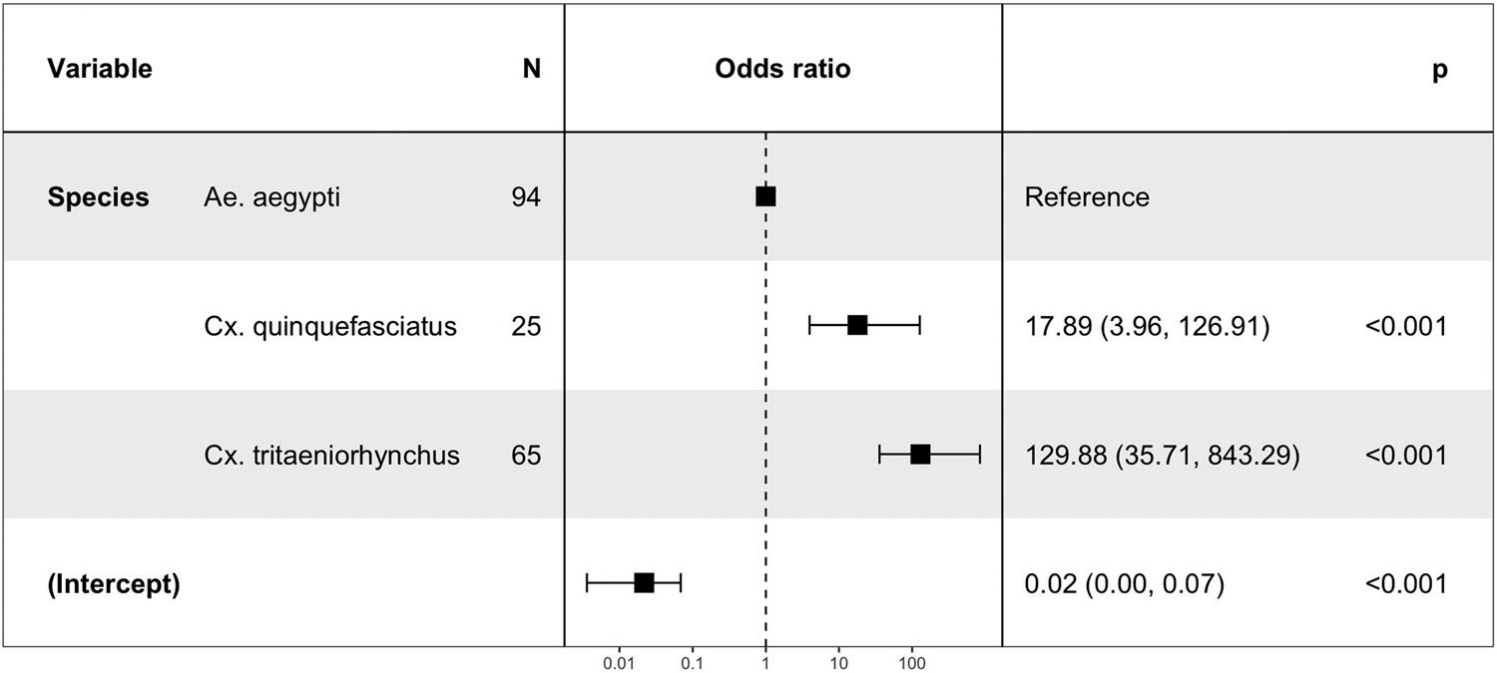
The risk factors for mosquito transmission.

In each additional day post-exposure, the chance of dissemination increases (OR = 1.06, *P* = 0.01) (Figure 5). DKV is more likely to be disseminated by *Cx. tritaeniorhynchus* (OR = 5.49, *P* < 0.001) than by *Ae. aegypti* and less likely to be disseminated by *An. gambiae* (OR = 0.06, *P* < 0.001) (Figure 5). Both *Culex* species were more likely to transmit DKV (Figure 6). The interaction terms between species and days were not significant for dissemination and transmission (*P* > 0.1). The day’s effect was not significant for transmission (*P* = 0.15).

## Discussion

DKV is a new mesonivirus species firstly reported in Senegal, West Africa, from several arthropods species, which include mosquitoes from the *Culex*, *Aedes*, *Anopheles*, and *Mansonia* genera [16]. Even if some studies have also described mesoniviruses infections in mosquitoes with significant public health impact [1,2,6], there is very little knowledge on their fitness in vectors. To our knowledge, this study is the first assessment of the possibility of transmission of viruses belonging to this group by mosquito saliva. A previous study only reported RNA detection/sequencing of other ISVs from salivary glands [19].

Of the anthropophilic mosquito species tested, the *Culex* mosquitoes were the most competent, followed by *Ae. aegypti* (Fisher’s exact test: *P* = 0.005 and Binary regression logistic: *P* < 0.001). *An. gambiae* populations were largely incompetent. *Cx. tritaeniorhynchus* and *Cx. quinquefasciatus* were the most susceptible to infection and were able to transmit DKV strain at all dpi tested. Both of these anthropophagic species are common and abundant in domestic environments where *Cx. quinquefasciatus* lays their eggs on artificial breeding sites (polluted water, septic tanks, etc.) [20] seldom colonized by *Cx. tritaeniorhynchus* which is often found in nearby ponds and rice fields [21]. The *Cx. quinquefasciatus* population used in this study has been proven competent for Rift Valley Fever virus (RVFV) [22]. Both *Culex* species are vectors of many other arboviruses, including West Nile, Japanese encephalitis viruses [23,24] and RVFV.

Our *An. gambiae* population was not competent for DKV strain. Mosquitoes appeared infected initially, but the virus disappeared by 4 dpi. Although the infection recrudesced at 15 dpi (to 16% infected) and disseminated at 20 dpi, this is too late to be epidemiologically relevant given the short lifespan of the mosquitoes in nature [25]. Recrudescence may have been a result of a delayed immune response in some individuals or a long eclipse phase followed by virus replication [26]. *An. gambiae* is a poor vector of arboviruses, with the exception of O’nyong nyong fever virus [27,28].

*Aedes aegypti* mosquitoes were able to transmit DKV. Infection rates decreased over the first 3 days after the infected bloodmeal was digested. Evidence of dissemination appeared, however, on the first dpi. The virus did not fully disseminate until 15 dpi with transmission rates of 9.09%. *Ae. aegypti* is an anthropophilic species but also biting a wide range of vertebrates [29,30], fully adapted to the human environment [31], abundant, worldwide distributed, active year-round because of its association with artificial breeding sites. These vectorial capacity characteristics could make it – as it is for arboviruses like the dengue [32], yellow fever [33], zika [34] and chikungunya viruses [35] – an efficient vector of DKV in case that a second host would involve, while its transmission rate is low. Indeed it has been demonstrated with the yellow fever virus that an incompetent *Ae. Aegypti* population (transmission rate of 7%) can support an epidemic transmission in the presence of high population density [36].

Our results showed that DKV viral particles remain infectious in anthropophilic mosquitoes’ saliva. One should note that DKV transmission to human or another vertebrate could occur during feeding, even if ISVs are characterized by their incapacity to replicate in vertebrate cells. The ISVs host range restriction could occur at different steps of the viral cycle, as Junglen et al. [37] showed a blocking at both attachment/entry as well as at the assembly /release level in vertebrate cells. The restriction mechanisms are still not very well understood even if it was reported that ISVs are more sensitive to high-temperature effects than the arboviruses [38]. It was then observed for DKV in vitro replication, which was impacted by thermal conditions [16]. It is also pointed out that the innate immune system in vertebrate cells can strongly restrict ISVs replication as described by Tree et al. [39]. In this study, knockdown of some pattern recognition receptors led to the replication of the ISV Kamiti River virus in both Vero and A549 cells, suggesting to that several of these receptors are important in the detection and control of ISVs in vertebrate cells.

Some studies showed evolutionary relationship between ISVs and arboviruses, suggesting that arboviruses could have been ISVs that later acquired the ability to expand their host-range to also include vertebrates [40]. Mesoniviruses share structural and genetic similarities to other vertebrates replicating Nidovirales [6]. Previous phylogenetic studies suggested that the coronaviruses and other viruses of the order may have evolved in arthropods [1,4]. The evolutionary process from ISVs to, at least, dual host viruses probably require a long period of time for adaptation before the occurrence of an ancestral host switching.

This study is the first to evaluate the vector competence of mosquitoes for DKV. Furthermore, using C6/36 cell culture, we demonstrated that the mosquitoes produced infectious virus particles in their saliva. This is an important point because, until now, vertical transmission was thought to be the main form of MESOV propagation [10,41–43]. This transmission modal had led previous studies on mesonivirus to use intrathoracic inoculation as infection route [44]. However recent studies using the Negev and Eilat viruses showed that they successfully infected adult mosquitoes fed with infectious blood [45,46].

Our work highlights the potential transmission of DKV to vertebrate through anthropophilic mosquito biting. Only the main arboviruses vectors were DKV competent while the malaria vector *An. gambiae* was not. The possibility of horizontal DKV transmission proved in this study highlights that DKV crosses all compartments within mosquito-like arboviruses. This shows potential interaction between DKV and arboviruses that can lead to different issues. For instance, it has been shown that dual viral infections in mosquito can alter viral infectivity [47] and ISVs are more and more considered as potential disease control agents in vector populations [40,48]. Virus restriction phenotypic could be more complex as Parry and Asgari [49] highlighted that restriction of Dengue virus replication in *Ae.aegypti* mosquitoes could be hindered by interactions between Aedes anphevirus (AeAV) a novel ISV and the endosymbiotic bacterium *Wolbachia pipientis*. In contrast, Zhang et al. [50] pointed out that Cell fusing agent virus, another ISV, favour Dengue virus replication in *Ae.aegypti* cell lines. Based on these considerations, the issue of DKV interactions with arboviruses circulating in our subregion requires further studies. *Ae. aegypti* could be a good target for co-infection studies involving mesonivirus and arboviruses with real impact on public health due to the important role that the species is playing as a vector of major arboviruses of public health interest. Moreover, DKV could be a good model to study host switching from naturally infected mosquitoes to vertebrate organisms.
